# Prospective study validating a multidimensional treatment decision score predicting the 24-month outcome in untreated patients with clinically isolated syndrome and early relapsing–remitting multiple sclerosis, the ProVal-MS study

**DOI:** 10.1186/s42466-024-00310-x

**Published:** 2024-03-07

**Authors:** Antonios Bayas, Ulrich Mansmann, Begum Irmak Ön, Verena S. Hoffmann, Achim Berthele, Mark Mühlau, Markus C. Kowarik, Markus Krumbholz, Makbule Senel, Verena Steuerwald, Markus Naumann, Julia Hartberger, Martin Kerschensteiner, Eva Oswald, Christoph Ruschil, Ulf Ziemann, Hayrettin Tumani, Ioannis Vardakas, Fady Albashiti, Frank Kramer, Iñaki Soto-Rey, Helmut Spengler, Gerhard Mayer, Hans Armin Kestler, Oliver Kohlbacher, Marlien Hagedorn, Martin Boeker, Klaus Kuhn, Stefan Buchka, Florian Kohlmayer, Jan S. Kirschke, Lars Behrens, Hanna Zimmermann, Benjamin Bender, Nico Sollmann, Joachim Havla, Bernhard Hemmer, the ProVal-MS study group, Ansgar Berlis, Benedikt Wiestler, Tania Kümpfel, Klaus Seelos, Jutta Dünschede, Roswitha Kemmner, Meinrad Beer, Jennifer Dietrich, Jonas Schaller

**Affiliations:** 1 Department of Neurology and Clinical Neurophysiology, Medical Faculty, University of Augsburg, Stenglinstrasse 2, 86156 Augsburg, Germany; 2 Institute of Medical Information Processing, Biometry, and Epidemiology, Faculty of Medicine, LMU Munich, Munich, Germany; 3 Department of Neurology, School of Medicine, Technical University of Munich, Klinikum rechts der Isar, Munich, Germany; 4 Department of Neurology and Stroke, and Hertie-Institute for Clinical Brain Research, Eberhard-Karls University of Tübingen, Tübingen, Germany; 5 Department of Neurology, University Hospital Ulm, Ulm, Germany; 6 Institute of Clinical Neuroimmunology, LMU Hospital, Ludwig-Maximilians-Universität München, Munich, Germany; 7 Medical Data Integration Center, University Hospital, LMU Munich, Munich, Germany; 8 IT-Infrastructure for Translational Medical Research, University of Augsburg, Augsburg, Germany; 9 Medical Data Integration Center, Institute of Digital Medicine, University Hospital Augsburg, Augsburg, Germany; 10 Medical Data Integration Center, Medical Center rechts der Isar, School of Medicine, Technical University of Munich, Munich, Germany; 11 Heidelberg Institute for Theoretical Studies (HITS), Heidelberg, Germany; 12 Institute of Medical Systems Biology, Ulm University, Ulm, Germany; 13 Institute for Translational Bioinformatics, University Hospital Tübingen, Tübingen, Germany; 14 Department of Computer Science, University of Tübingen, Tübingen, Germany; 15 Institute for Bioinformatics and Medical Informatics, University of Tübingen, Tübingen, Germany; 16 Institute for Artificial Intelligence and Informatics in Medicine, Medical Center rechts der Isar, School of Medicine, Technical University of Munich, Munich, Germany; 17 Bitcare GmbH, Munich, Germany; 18 Department of Diagnostic and Interventional Neuroradiology, School of Medicine, Klinikum rechts der Isar, Technical University of Munich, Munich, Germany; 19 Diagnostic and Interventional Neuroradiology, Faculty of Medicine, University of Augsburg, Augsburg, Germany; 20 Institute of Neuroradiology, LMU Hospital, Ludwig-Maximilians-Universität München, Munich, Germany; 21 Department of Diagnostic and Interventional Neuroradiology, University Hospital Tübingen, Tübingen, Germany; 22 Department of Diagnostic and Interventional Radiology, University Hospital Ulm, Ulm, Germany; 23 Munich Cluster for Systems Neurology (SyNergy), Munich, Germany

**Keywords:** Multiple sclerosis, Clinically isolated syndrome, Prospective cohort, Treatment decision, Data integration, Validation, Routine data, Clinical trial

## Abstract

**Introduction:**

In Multiple Sclerosis (MS), patients´ characteristics and (bio)markers that reliably predict the individual disease prognosis at disease onset are lacking. Cohort studies allow a close follow-up of MS histories and a thorough phenotyping of patients. Therefore, a multicenter cohort study was initiated to implement a wide spectrum of data and (bio)markers in newly diagnosed patients.

**Methods:**

ProVal-MS (Prospective study to validate a multidimensional decision score that predicts treatment outcome at 24 months in untreated patients with clinically isolated syndrome or early Relapsing–Remitting-MS) is a prospective cohort study in patients with clinically isolated syndrome (CIS) or Relapsing–Remitting (RR)-MS (McDonald 2017 criteria), diagnosed within the last two years, conducted at five academic centers in Southern Germany. The collection of clinical, laboratory, imaging, and paraclinical data as well as biosamples is harmonized across centers. The primary goal is to validate (discrimination and calibration) the previously published DIFUTURE MS-Treatment Decision score (MS-TDS). The score supports clinical decision-making regarding the options of early (within 6 months after study baseline) platform medication (Interferon beta, glatiramer acetate, dimethyl/diroximel fumarate, teriflunomide), or no immediate treatment (> 6 months after baseline) of patients with early RR-MS and CIS by predicting the probability of new or enlarging lesions in cerebral magnetic resonance images (MRIs) between 6 and 24 months. Further objectives are refining the MS-TDS score and providing data to identify new markers reflecting disease course and severity. The project also provides a technical evaluation of the ProVal-MS cohort within the IT-infrastructure of the DIFUTURE consortium (Data Integration for Future Medicine) and assesses the efficacy of the data sharing techniques developed.

**Perspective:**

Clinical cohorts provide the infrastructure to discover and to validate relevant disease-specific findings. A successful validation of the MS-TDS will add a new clinical decision tool to the armamentarium of practicing MS neurologists from which newly diagnosed MS patients may take advantage.

*Trial registration* ProVal-MS has been registered in the German Clinical Trials Register, `Deutsches Register Klinischer Studien` (DRKS)—ID: DRKS00014034, date of registration: 21 December 2018; https://drks.de/search/en/trial/DRKS00014034

**Supplementary Information:**

The online version contains supplementary material available at 10.1186/s42466-024-00310-x.

## Background

Multiple Sclerosis (MS) is a chronic inflammatory disease of the central nervous system (CNS) with considerable impact on individual health, but also socioeconomic factors [24]. An estimation of the most likely disease course is not possible with certainty at initial diagnosis, as the clinical course of MS can vary from benign to highly aggressive. This is also reflected by heterogeneity in clinical and imaging phenotypes. [8, 9] In recent years, enormous progress has been made in understanding the immunopathogenesis and its relationship with genetic and environmental factors, which can influence disease risk and possibly also disease progression [6, 14].

Increasingly, disease modifying therapies (DMTs) are available, which differ in mode of action, therapeutic efficacy, risk profile, route of administration, and treatment burden/monitoring requirements. Highly effective drugs may be associated with potentially severe risks. Therefore, therapy selection remains individualized based on the patient's disease status, presumed prognosis, and risk tolerance.

Differences in efficacy, monitoring requirements, and safety of DMTs have led to diverging therapeutic strategies: the so-called escalation approach compared to the "hit hard and early" approach. While the first approach may result in insufficient therapy, the latter may lead to overtreatment in patients not having a highly active disease course. The “wait and see” approach may also be considered as an option for a newly diagnosed patient. Prognostic research addresses this issue by establishing predictive markers of MS disease activity and progression being as simple, reproducible, and reliable as possible. This may help in establishing more targeted treatment strategies without unwarranted risk to the patient [5, 7, 18, 22]. Prognostic markers have already been identified at the group level. However, in summary, results to date indicate that a combination of potential prognostic markers provides a more accurate prognostic estimate than a single factor alone [19, 22]. This, however, requires models to estimate the individual risk and to consider the interaction between patient features and specific treatment options. Accordingly, these prediction tools are empirical models that quantify the effects of the combination of two or more predictive factors and attempt to estimate the likelihood of clinical disease activity or progression individually over a given time [21, 22]. However, it is important that these prognostic models are valid, warranting strict requirements for prognosis research, and that they are developed and validated using large, high-quality datasets with subjects who are representative of the population to which the model is later to be applied [22]. Deeply characterized cohorts are needed for prognosis research.

The aim of ProVal-MS is to contribute to the discovery and validation of novel predictive markers from routine university care for stratification of patients with respect to progression and response to treatment using a federated technical and organizational approach that stands for innovative data-driven medical research. Here we present the protocol of the ongoing ProVal-MS study.

## Methods

### Aim of the trial

The primary and secondary objective of ProVal-MS (German Clinical Trials Register-ID: DRKS00014034) is to validate discrimination and calibration of the Data Integration for Future Medicine MS-treatment decision score (DIFUTURE-TDS) on the 24 months outcome in early relapsing–remitting multiple sclerosis (RR-MS) and clinically isolated syndrome (CIS) patients, diagnosed within the last two years, using the area under the curve (AUC) of the receiver operating characteristics curve (ROC) and the calibration curve. The score supports clinical decision-making regarding the options of early (within 6 months after study baseline) platform medication (Interferon beta, glatiramer acetate, dimethyl/diroximel fumarate, teriflunomide), or no immediate treatment (> 6 months after baseline) of patients with early RR-MS and CIS by predicting the probability of new or enlarging lesions in cerebral magnetic resonance images (MRIs) between 6 and 24 months.

Patients starting on high efficacy drugs may not be included in the validation of the MS-TDS but are considered for secondary and exploratory endpoint analyses.

The MS-TDS has been created and internally validated by integration of retrospective routine clinical, imaging and laboratory data from 65 predictors (Additional file 1: Table S1) collected for deeply characterized 475 MS patients at the “Klinikum rechts der Isar, Technical University of Munich” [4]. To create the MS-TDS, a predictive random forest (RFs) model was implemented through transformation forests based on fully parameterized Cox proportional hazards models to deal with the interval-censored outcome [11, 15]. A benchmark study was performed for hyperparameter tuning and to choose the best performing model. In order to identify informative predictor variables, likelihood-based permutation variable importance measures (VIMPs) of this final model were used.[3, 12]. The five most important predictor variables with VIMP exceeding the VIMP of a random noise variable in the final model were, in descending order, treatment, periventricular lesions, total MRI lesions, CSF-specific oligoclonal bands, and relapses, item `any other symptom` [4].

The MS-TDS predicts the probability of new or enlarging lesions in cerebral magnetic resonance images (MRIs) between 6 and 24 months after the first MRI. Its cross-validated area under the curve (AUC) was 0.624 [4].

Further objectives are refining the MS-TDS score and providing data to identify new markers reflecting disease course and severity. The project also provides a technical evaluation of the ProVal-MS cohort within the IT-infrastructure of the DIFUTURE consortium (Data Integration for Future Medicine, www.difuture.de) and assesses efficacy of the data sharing techniques developed.

### Study description and study design

ProVal-MS (German Clinical Trials Register-ID: DRKS00014034) is a prospective, multi-center, non-interventional, diagnostic phase II cohort study in, at study inclusion, untreated patients with early RR-MS or CIS.

### Eligibility criteria

Patients aged 18–60 years and diagnosed with CIS (meeting the requirements of dissemination in space but not in time) or RR-MS according to the McDonald 2017 [23] diagnostic criteria at the time of diagnosis and before the initiation of any immunotherapy (except corticosteroids if the last treatment was more than 4 weeks before inclusion) are eligible to participate. Inclusion and exclusion criteria are summarized in Table 1.

**Table 1:** Inclusion and exclusion criteria for ProVal-MS

Inclusion criteria	CIS, with dissemination in space *or* relapsing remitting MS according to the 2017 McDonald criteria^a^ Diagnosed within the last two years Treatment naïve 18 until 60 years of age
Exclusion criteria	Under any administrative or legal supervision Unable to give informed consent, Previous treatment with disease modifying therapies (including corticosteroid therapy of relapses, if given less than four weeks prior baseline) Conditions/concomitant diseases making the patient non-evaluable for the primary endpoint (e.g., pre-existing neurological disease, systemic autoimmune diseases) Requirement for concomitant treatment that could bias primary evaluation Inability to meet specific protocol requirements (e.g., need for hospitalization, not able to read and understand the protocol), Patients directly involved in the conduct of the protocol: investigator or subinvestigator, research assistant, pharmacist, study coordinator, other staff or relative thereof Patient is uncooperative or has any condition that could make the patient potentially non-compliant to the study procedures Pregnant or breast-feeding women

^a^Thompson AJ, Banwell BL, Barkhof F, et al. Diagnosis of multiple sclerosis: 2017 revisions of the McDonald criteria. *The Lancet Neurology*. Feb 2018;17(2):162–173. 10.1016/S1474-4422(17)30470-2

### Sample size consideration for the minimal cohort size

At study start, a total of 250 patients were planned to be included to reach (e.g., after dropouts or protocol violations) at least 188 patients with available DIFUTURE-MS-TDS, which ensures sufficient power for validation. Its minimal sample size was determined by the requirement to reject the null-hypothesis (AUC ≤ 0.7) given the desired alternative (AUC = 0.8) on a 5% alpha level with power of at least 80%.

### Arms and interventions

Due to the non-interventional study design, treatment allocation or procedures, starting after written informed consent and recording of baseline data, is not part of the study and follows current clinical routine practice. ProVal-MS is a non-interventional study, study results are derived from the whole patient cohort without stratifying them to different observation groups. The cohort structure allows the definition of and comparison between subgroups.

### Outcome measures

The primary outcome of ProVal-MS is defined as achieving no new or enlarging T2-lesions (an area of hyperintensity on a T2-weighted MRI scan that is at least 3 mm in long axis) in cranial MRI between study month 6 and 24 (Yes, No).

MRI at month 6, is considered as `rebaseline` MRI after DMT initiation and is equally performed in untreated study participants. The MRI schedule is equal for all centres, according to the study protocol, essentially based on standard clinical practice in the participating centres. According to the study protocol, there is no MRI scheduled between month 6 and 24. Performing an MRI in case of a relapse is upon the decision of the treating physician. Patients reaching the primary endpoint already before the month 6 MRI, will be included in the analysis as patients without MRI activity in this period. The same applies for patients initiating a treatment or switching to another treatment between month 6 and 24. Any relapses occurring after the baseline and before the month 6 MRI will be considered for analysis in line with the MS-TDS, such as within 3 months from baseline, for validation purposes. The AUC is calculated by comparing the individual binary primary outcome with the individual DIFUTURE-MS-TDS score that considers the patient’s features at baseline and the therapy given. Calibration curves investigate the alignment of the 24-months success predicted from the DIFUTURE-MS-TDS with the 24-months outcome actually observed. The Brier Score summarizes the predictive quality of DIFUTURE-MS-TDS. Explorative analyses on the association between primary outcome, specific biomarkers, e.g. serum levels of Glial Fibrillary Acidic Protein (GFAP), neurofilament light chain (NfL), and others to be determined, not used for creating the MS-TDS [4], and treatments given are of further relevance.

Study procedures are undertaken with the understanding and after written consent of each subject, the study conforms with the World Medical Association Declaration of Helsinki. The study was approved by the local ethical review committees [University Hospital Augsburg (UKA), Ludwig-Maximilians-Universität (LMU) Hospital Munich, 18-0484; Klinikum rechts der Isar, Technical University of Munich (TUM), 323/18 S; Eberhard Karl University, Tübingen (UKT), 553/2018B02; Ulm University Medical Center (UKU), 238/19].

### Data collection and sharing

All ProVal-MS sites collect data under a common data model which follows current international documentation standards in MS. The sites use the local clinical data capture systems. The local medical Data Integration Centers (meDICs) transfer the data into the local study databases (local DIS, the open-source software *Data Integration System* [16]: https://www.bitcare.de/). DIS also captures data outside of the clinical routine (different questionnaires: GEMS, COVID-19, see Additional file 2: Table S2). It also includes a secure identity management component. All local data are stored and managed in the meDICs of each study site.

**Fig. 1:**
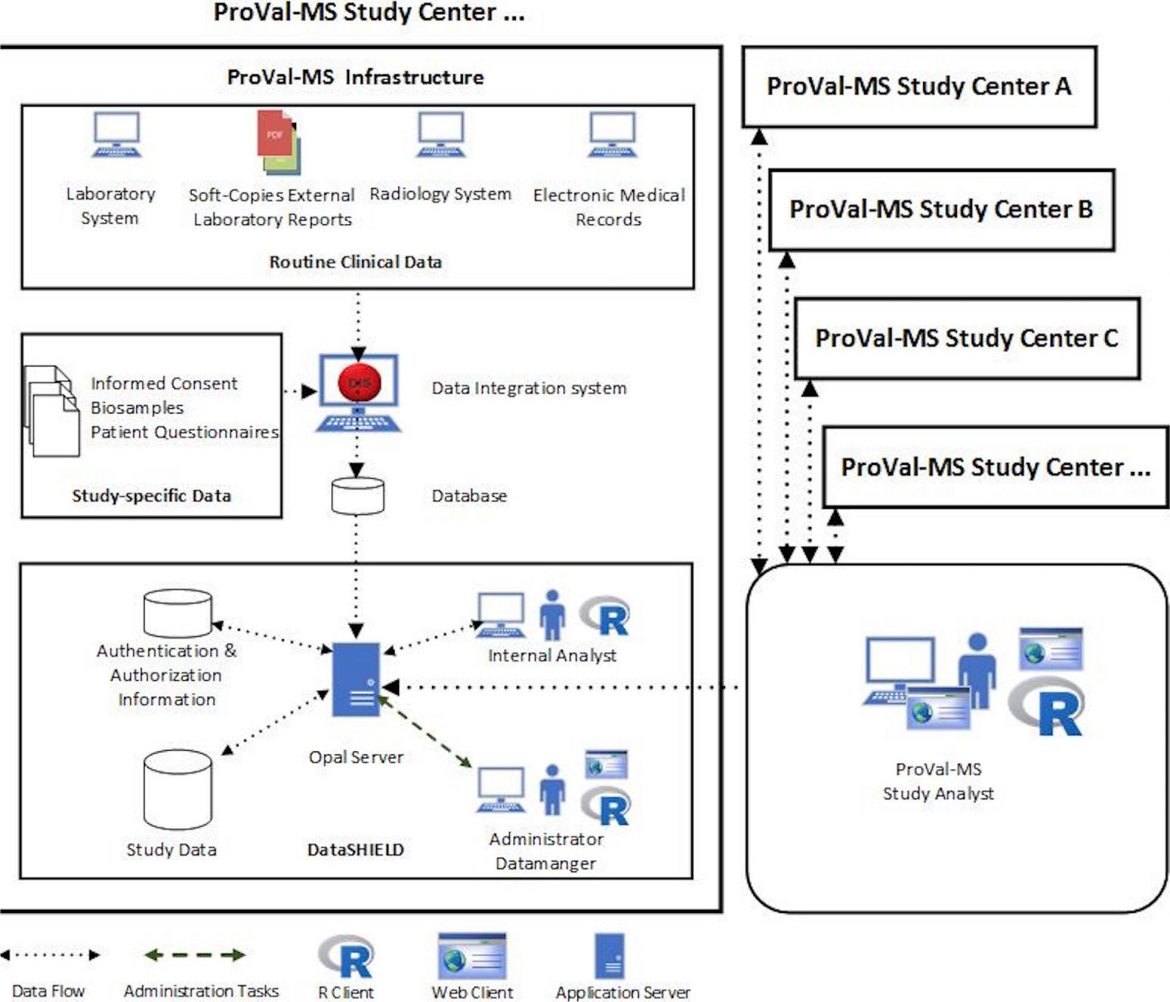
The multi-centric study infrastructure of ProVal-MS established at each study center. The routine clinical data is imported, and the study specific data is entered into the Data Integration System (DIS). The study data is made available in the DataSHIELD Opal server and accessible by the ProVal-MS study analyst to perform privacy preserving analysis using the R Client or Webclient (R, free software environment for statistical computing and graphics)

Inspite the federated datastructure, patients also gave consent to pool their data across sites. For the primary analysis, pooled data as well as a federated strategy will be used and compared. The principle technical goal of the study is to establish and validate the federated analysis strategy by DataSHIELD. DataSHIELD [26] provides a framework for distributed analysis that preserves privacy between the different centres (individual patient features will not be recognizable outside the center where the patient is treated). Figure 1 shows the cohort’s multi-centric federated study infrastructure.

### Clinical and paraclinical assessments

Within the consented data model (clinical and paraclinical data and metadata), a mandatory core dataset and an extended dataset were defined. All clinical data were collected in a harmonized and standardized manner. All predictors of the MS-TDS (Additional file 1: Table S1) are obligatory in the ProVal-MS study except CSF. Since patients included must have been diagnosed within the last two years and CSF is routinely taken for diagnosis, if patients consent, we expect to have CSF parameters in more than 95% of study participants for TDS-MS validation.

Patients have four mandatory study visits: Screening, baseline, visit at month 6, and at month 24. Visit at month 12 is not mandatory. Furthermore, for long-term follow-up of the cohort until year 5 after baseline, annual visits (month 36, 48, and 60) were implemented, after obtaining informed consent (study protocol amendment approved by the local ethical review committees).

Each mandatory visit collects the following core clinical parameters: Relapse activity and outcome, standard neurological examination, Expanded Disability Status Scale (EDSS; Version 04/10.2, https://www.nationalmssociety.org/NationalMSSociety/media/MSNationalFiles/Brochures/10-2-3-29-EDSS_Form.pdf), 9-hole-peg test, 25-foot walk test, low contrast visual acuity (2.5% sloan charts), oral Symbol Digit Modality Test (SDMT), questionnaires for depression (Beck depression inventory II, BDI-II), the GEM Environmental Questionnaire (at least once in the study period), fatigue (Fatigue Scale for Motor and Cognitive Functions, FSMC). A quality-of-life questionnaire (short form 36, SF36) and the Paced Auditory Serial Addition Test (PASAT) was optional at each visit.

In terms of paraclinical parameters, obligatory tests are cerebral and spinal MRI, routine laboratory data (see Additional file 3: Table S3; depending on the DMT, additional parameters may be analyzed at the discretion of the treating physician), and for secondary and exploratory analyses a collection of biosamples used for multi-OMICs analyses and biomarker assays, e.g., GFAP, NfL, and others to be determined, at baseline, months 6 and 24 drawn as part of the routine diagnostic procedure. Optical coherence tomography (OCT), laboratory analyses of cerebrospinal fluid, evoked potentials (visual, somatosensory, motor evoked potentials, VEP, SEP, MEP) are optional.

The prescription of disease modifying therapies (DMTs) or the option of not to treat is entirely the responsibility of the treating physician (investigator) following the standard of care. Treatments, started after baseline visit, and concomitant medication are recorded.

Paper-based source documents (including narratives) are used for monitoring. Parameters are collected during study visits which are scheduled for baseline, month 6 (−4/ + 8 week-interval) and month 24 (± 8 week-interval), performed according to the standard clinical practice with additional blood sampling. An additional visit after 12 months (± 8 weeks) is non-mandatory. Additional file 2: Table S2 provides the overall time schedule and study procedures. By study protocol amendments, month 6-visit is scheduled 6 months after treatment initiation, if started within 6 months after baseline, extending the study period in select patients up to 30 months. No additional invasive procedures that are not part of the routine practice are performed.

### Magnetic resonance imaging

Imaging protocols for cerebral and spinal MRI were highly standardized across all participating centres with the prerequisite to use the same scanner, at least from month 6 (visit 2) on. Both cerebral follow-up scans (month 6 and 24) have to be performed at the same 3.0 Tesla (T) scanner with the same coils and protocols to ensure longitudinal comparability. Spinal follow up MRIs can be performed at 1.5T or 3.0T but must also be obtained at one single scanner. Baseline cerebral and spinal MRI may be performed at any 1.5T or 3.0T scanner; however, if possible, it is recommended to use the same scanner as for follow-up scans. Pulse sequence protocols are shown in Table 2. In each centre, responsibilities to evaluate MRI scans have been assigned to neuroradiologists involved in the study. Cranial and spinal MRI scans are analyzed manually at each study centre. Structured reporting software (mintLesion™, LMU, UKT) and longitudinal subtraction to assess new brain lesions by commercially available software (Philips Longitudinal Brain Imaging, LoBi, or Siemens Healthineers syngo.via, UKA, TUM) is used in the study centres according to local practise. Additionally, pseudonymized cranial MRI scans are analysed centrally (TUM; J.S.K) by an in-house developed longitudinal subtraction algorithm [2] and an automated lesion segmentation software (lesion segmentation tool–artificial intelligence) [25] and results are compared with the results obtained by manual analysis. Discrepancies between these methods, if existing, are resolved centrally by personal comparison, to reach a final consensus in each patient. The consented final data are used as endpoints for the study.

**Table 2:** MRI protocol for cranial and spinal MRI

Cranial MRI	Fluid-Attenuated Inversion Recovery (FLAIR), 3D 1 mm isotropic or less Magnetization Prepared Rapid Acquisition Gradient Echo (MPRAGE, or other 3D T1-weighted gradient echo sequence) 1 mm isotropic or less T2 Turbo Spin Echo/Fast Spin Echo (TSE), 2D axial, 5 mm or less (alternative: 3D T2), Diffusion-Weighted Imaging (DWI), axial, 5 mm or less Optional: MPRAGE (or other 3D T1-weighted sequence) + contrast agent, T1 Spin Echo (SE) + contrast agent Double Inversion Recovery (DIR) 1.0–1.5 mm isotropic T2 TSE, 2D sagittal
Spinal MRI	1.5 T: Sagittal T2 TSE 2.5 mm, axial T2 TSE 4 mm or less (covering the whole spinal cord) 3 T: Sagittal T2 TSE 2.5 mm or less, axial T2 TSE 4 mm or less (covering the whole spinal cord); optional: T1 sagittal −/ + contrast agent, T1 axial −/+ contrast agent

### Optical coherence tomography

A standardized OCT protocol was agreed upon and established across sites. The following 3 parameters are measured: (1) The thickness of the peripapillary retinal nerve fiber layer (pRNFL) in µm. (2) The thickness (µm) or (3) volume (mm^3^) of the different retinal layers around the fovea. OCT data will be reported according to the APOSTEL [1] and OSCAR-IB [20] recommendations.

### Biosamples

Blood samples for DNA, RNA/plasma, serum (all mandatory) and peripheral blood mononuclear cells (optional) are collected at all mandatory visits. A central biosample management facility is provided by Technical University of Munich responsible for processing, analysis, and long-term storage to ensure consistent data quality. Sampling is performed with pre-labeled, barcoded tubes in sampling kits. The pseudonym on each sampling kit will be linked to the patient pseudonym in the local study database. Thus, all biosample-related data will be protected by two-step pseudonymization at all sites, ensuring data privacy. Sampling follows the same procedure as established in the Horizon 2020 EU Project Multiple MS cohort [10]. By study protocol amendment, a COVID-19 patient questionnaire and analysis of biosamples regarding COVID-19 immunoreactivity were implemented.

### Data evaluation/statistics

DataSHIELD also allows to perform data quality checks over the distributed data. This process is similar to query process in clinical studies: A quality assessment script is distributed over the centers, each center creates its own data quality report and learns on data items which do not comply with the quality criteria established for a project. The curated data can be used to perform local research projects as well as projects over all five centers. Descriptive statistics can also be implemented under a distributed privacy-protecting strategy. Within the Medical Informatics Initiative (MII), there is an active development of algorithms applicable within DataSHIELD and allowing privacy protected distributed machine learning (privateaim.de).

In order to assess the primary endpoints, the ROC as well as AUC are calculated using distributed as well as centralized approaches. Calibration curves investigate the alignment of the 24-months success predicted from the DIFUTURE-MS-TDS with the 24-months outcome actually observed. The Brier Score summarizes the predictive quality of DIFUTURE-MS-TDS. Explorative analyses on the association between primary outcome, specific biomarkers, e.g. serum levels of GFAP, NfL, and others to be determined, not used for creating the MS-TDS [4] and treatments given are of further relevance.

### Contacts for sponsors and collaborators, investigators

The ProVal-MS study reported here was established within the audited use case of the DIFUTURE (www.difuture.de) consortium as part of the German MII. MII is establishing meDICs at many German university hospitals. The following study centers participate together with their meDICs: Department of Neurology and Clinical Neurophysiology, University Hospital Augsburg (UKA; Prof. Dr. Antonios Bayas, Prof. Dr. Markus Naumann); Department of Neurology, Klinikum rechts der Isar, Technical University of Munich (TUM; Prof. Dr. Bernhard Hemmer); Institute of Clinical Neuroimmunology, LMU Hospital, Ludwig-Maximilians-Universität Munich (LMU; Prof. Dr. Martin Kerschensteiner; PD Dr. Joachim Havla); Department of Neurology and Hertie Institute for Clinical Brain Research, Eberhard Karl University, Tübingen (UKT; Prof. Dr. Ulf Ziemann; PD Dr. Markus C. Kowarik), Department of Neurology, Ulm University Medical Center (UKU; Prof. Dr. Hayrettin Tumani, PD Dr. Makbule Senel)) within and associated with the DIFUTURE consortium.

Responsibilities have been deputed as follows: coordinating investigator: Prof. Dr. Bernhard Hemmer; Biometrician: Prof. Dr. Ulrich Mansmann; IT coordinator: Prof. Dr. Oliver Kohlbacher; central quality assurance: Marlien Hagedorn.

To ensure compliance with the rules of good clinical practice, this study is being monitored by a steering committee (Prof. Dr. Bernhard Hemmer, Prof. Dr. Martin Kerschensteiner, Prof. Dr. Ulf Ziemann, Prof. Dr. Antonios Bayas, Prof. Dr. Ulrich Mansmann, Prof. Dr. Oliver Kohlbacher, Prof. Dr. Klaus A. Kuhn). Additional investigators and collaborators can be found in the authors and study group list.

This work as part of the DIFUTURE project has been funded by the German Ministry of Education and Research (Bundesministerium für Bildung und Forschung, BMBF), Grant 01ZZ1804[A-I].

### Perspective

The highly variable and apparently unpredictable MS disease course makes the assessment of the individual prognosis difficult or rather impossible. According to the treat-to-target algorithm, the use of DMTs is dependent on individual disease activity, supported by disease prognosis markers based on findings in larger MS cohorts [13]. Having reliable predictive markers would enable the clinician to tailor treatment according to the individual prognosis avoiding under- and overtreatment with the danger of accumulating disability or avoidable hazard to the patient.

As the study was initiated as use case within the MII, ProVal-MS has several objectives; one is to show that it is feasible to use data collected in the routinely used clinical data capture systems for building clinical cohorts with high relevance for clinical research. Usually, data for clinical studies is collected within (electronic) case report forms (eCRFs) requiring clinicians or study nurses to collect relevant data twice, once in the standard patient record and once in the eCRF. However, for many studies there are large intersections between the data collected in the clinical IT systems and the data needed for research purposes. Entering those into two separate systems is time- and resource-consuming and prone to discrepancies resulting in data entry errors. Thus, for ProVal-MS routinely collected data is collected with high quality (changing processes within the clinic as well as the clinical IT) and exported directly from the source systems. The local practice changes were driven by a common MS core dataset that harmonized data structures at the participating sites. Local changes included adaption or programming of local entry formats or mapping the source information into the core dataset. This enabled an easy transfer of high-quality routine data to the study database where it was enriched with study-specific data captured outside of the clinical routine. This effort also increased the local standard of routine documentation supporting future research based on structured routine data. As in our study, to allow for timely results while complying with data protection regulations, methods of distributed analysis can be applied using e.g. DataSHIELD [26] as a framework for privacy-preserving distributed analysis of sensitive data like routine data of MS patients, which may be analyzed locally but not shared between sites. This concept will be applied and validated during the analysis in ProVal-MS. While the preparatory work needed to harmonize data, making them available from routine IT systems is considerable, the resulting datasets enable the full potential of routine data for high-quality clinical research. The concepts for data structure and integration developed within DIFUTURE are universal and can be applied to more sites therefore providing an easier introduction into providing data for research.

In a recent Cochrane Review, all multivariable prognostic model developments and validations published to date for quantifying the risk of clinical disease progression, worsening, and activity in MS were identified and summarized. Only 2 of 75 models were shown to be validated in external cohorts multiple times, which, however, is formally the most stringent quality standard of prognostic research. Also, the studies reviewed differed in terms of clinical characteristics of the patient cohorts, definition of predictors, and outcome parameters, which made comparability difficult [17]. Overcoming this, ProVal-MS is a validation study for the DIFUTURE-MS-TDS emerging from a combined analysis of existing data in a separately conducted retrospective cohort study, thus validating a predictive model in a different prospective cohort using clinical routine data.

Besides the overall strategy of data standardization across centers, local data integration and decentralized data analysis via distributed computing, ProVal-MS has several unique features compared to other prospective cohorts. The study uses high-resolution standardized 3T imaging of the brain and the entire spinal cord (1.5 or 3T) at months 6 and months 24 to detect inflammatory disease activity. Besides the large number of clinical parameters, paraclinical parameters such as evoked potentials and OCT are recorded at diagnosis and during the disease course.

In conclusion, ProVal-MS as a prospective multi-center cohort study will add important in-depth data for MS prognosis research. Furthermore, it should prove that with the appropriate IT infrastructure and data harmonization, it is feasible to use data collected in the routinely used clinical data capture systems for clinical research. Data resulting from this may in turn support clinicians in patient counseling and treatment.

### Supplementary Information

**Additional file 1: Table S1.** Predictors of the MS-treatment decision score.

**Additional file 2: Table S2.** Study procedure overview.

**Additional file 3: Table S3.** Routine laboratory parameters.

## Data Availability

All data and materials reported are available from the corresponding author upon reasonable request.

## Abbreviations

AUC: Area under curve
BDI-II: Beck depression inventory II
CIS: Clinically isolated syndrome
CNS: Central nervous system
DIFUTURE: Data integration for future medicine
DIS: Data integration system
DMTs: Disease modifying therapies
eCRFs: Electronic case report forms
EDSS: Expanded disability status scale
FSMC: Fatigue scale for motor and cognitive functions
IT: Information technology
meDIC: Medical data integration centers
MEP: Motor evoked potentials
MII: Medical informatics initiative
MRI: Magnetic resonance imaging
MS: Multiple sclerosis
OCT: Optical coherence tomography
PASAT: Paced auditory serial addition
pRNFL: Peripapillary retinal nerve fiber layer
ProVal-MS: Prospective study to validate a multidimensional decision score to predict treatment outcome at 24 months in untreated patients with clinically isolated syndrome or early RR-MS
ROC: Receiver operating characteristics
RR: Relapsing–remitting
RR-MS: Relapsing–remitting multiple sclerosis
SDMT: Symbol digit modality test
SEP: Somatosensory evoked potentials
SF36: Short form 36
T: Tesla
TDS: Treatment decision score
VEP: Visual evoked potential
