# Supplementary material for: Prospective study validating a multidimensional treatment decision score predicting the 24-month outcome in untreated patients with clinically isolated syndrome and early relapsing–remitting multiple sclerosis, the ProVal-MS study
Source: Neurol Res Pract. 2024 Mar 7;6:15. doi: 10.1186/s42466-024-00310-x (PMC10918966; doi:10.1186/s42466-024-00310-x)
Supplement: Supplementary file 1 — Additional file 1: Table S1. Predictors of the MS-treatment decision score. [file 42466_2024_310_MOESM1_ESM.docx]

Table S1. Predictors of the MS-treatment decision score

|  | Predictors |
| --- | --- |
| Sex |  |
| Age (years) |  |
| BMI (kg/m^2^) | BMI (kg/m^2^)  Height (cm)  Weight (kg) |
| Smoking |  |
| Diagnosis | Diagnosis at baseline  Time since diagnosis at baseline (days) |
| First symptom | Numbness  Other cranial nerve symptom  Paresis  Optic neuritis  Any other symptom |
| Relapses (± 3 months from baseline) | Numbness  Other cranial nerve symptom  Paresis  Optic neuritis  Any other symptom |
| Number of relapses during 3 years before baseline |  |
| EDSS/functional score | Total  Pyramidal  Cerebellar  Brainstem  Sensory  Bowel and bladder  Visual  Cognitive  Ambulation |
| FSMC | Fatigue cognitive  Fatigue motor  Total |
| MSFC | Nine-Hole Peg test result - hand/arm  25-Foot Walk test result - ambulation |
| BDI-II - depression |  |
| First cMRI | Total lesions count  Periventricular lesions present  Subcortical/unspecific lesions present  Juxtacortical or cortical lesions present  lnfratentorial lesions present |
| CSF | Leucocyte count (cnt/mcl)  Glucose (mg/dl)  Total protein (mg/dl)  Albumin quotient (x10^-3^)  CSF-specific oligoclonal bands (no/borderline/yes)  lgG Quo/Alb Quo (lgG-index)  lgM Quo/Alb Quo (lgM-index)  lgA Quo/Alb Quo (lgA-index) |
| Laboratory - blood analysis | Basophils (%)  Bilirubin (mg/dl)  Blood urea nitrogen (mg/dl)  Eosinophils (%)  Erythrocytes (cnt/pl)  GOT (ASAT) (U/l)  GPT (ALAT) (U/l)  Haematocrit (%)  Haemoglobin (g/dl)  Leucocytes (cnt/nl)  Lymphocytes (%)  MCH (pg)  MCHC (g/dl)  MCV (fl)  Monocytes (%)  Neutrophils (%)  Thrombocytes (cnt/nl)  TSH (mclU/ml) |

ASAT, aspartate transaminase; ALAT, alanine transaminase; BDI, Beck Depression lnventory; BMI, body mass index; cMRI, cerebral magnetic resonance images; CSF, Cerebrospinal fluid; EDSS, Expanded Disability Status Scale; FSMC, Fatigue Scale for Motor and Cognitive Functions; GOT, glutamic oxaloacetic transaminase; GPT, glutamic-pyruvic transaminase; lgA, lmmunoglobulin A; lgG, lmmunoglobulin G; lgM, lmmunoglobulin M; IQR, interquartile range; MCH, mean corpuscular haemoglobin; MCHC, mean corpuscular haemoglobin concentration; MCV, mean corpuscular volume; MSFC, MS functional composite; Quo, quotient; TSH, thyroid-stimulating hormone.
