# Supplementary figures and images for: Prospective study validating a multidimensional treatment decision score predicting the 24-month outcome in untreated patients with clinically isolated syndrome and early relapsing–remitting multiple sclerosis, the ProVal-MS study
Source: Neurol Res Pract. 2024 Mar 7;6:15. doi: 10.1186/s42466-024-00310-x (PMC10918966; doi:10.1186/s42466-024-00310-x)

Table S2. Study procedure overview
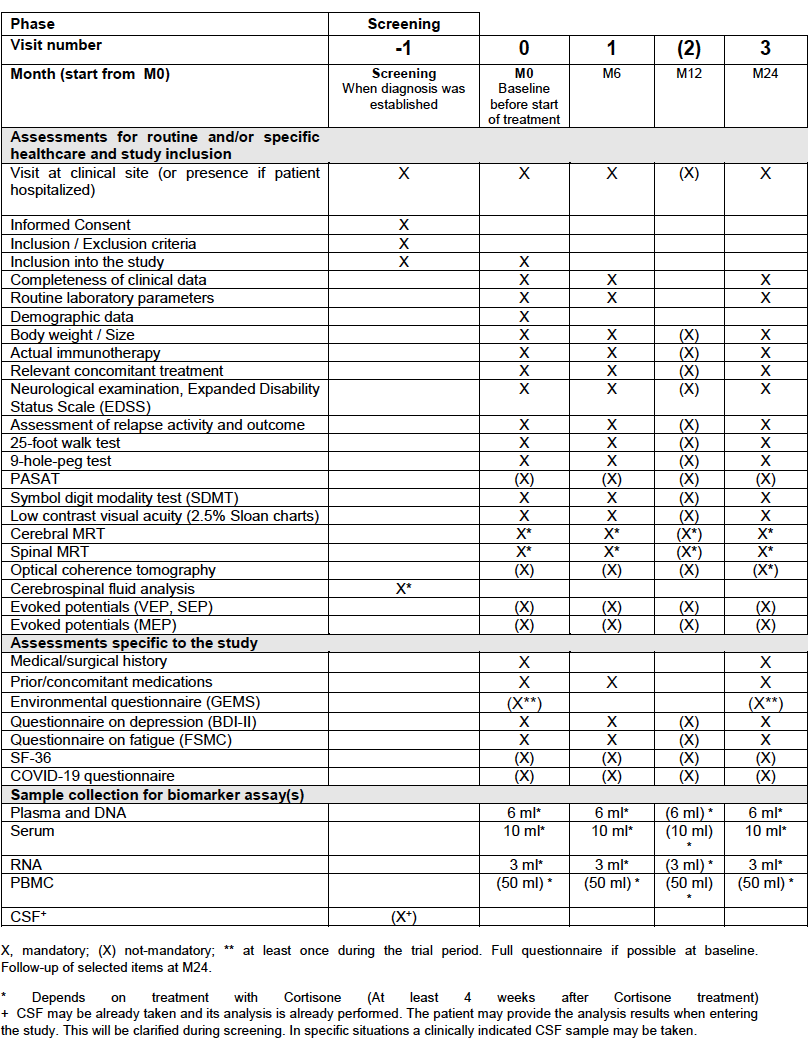

Supplement: Supplementary file 2 — Additional file 2: Table S2. Study procedure overview. [file 42466_2024_310_MOESM2_ESM.docx]
