# Supplementary material for: Prospective study validating a multidimensional treatment decision score predicting the 24-month outcome in untreated patients with clinically isolated syndrome and early relapsing–remitting multiple sclerosis, the ProVal-MS study
Source: Neurol Res Pract. 2024 Mar 7;6:15. doi: 10.1186/s42466-024-00310-x (PMC10918966; doi:10.1186/s42466-024-00310-x)
Supplement: Supplementary file 3 — Additional file 3: Table S3. Routine laboratory parameters. [file 42466_2024_310_MOESM3_ESM.docx]

Table S3. Routine laboratory parameters

| \| Creatinine \|  \| \| --- \| --- \| |
| --- | --- | --- |
| Urea |
| Total bilirubin |
| \| Gamma-glutamyltransferase \|  \| \| --- \| --- \| |
| Alanine aminotransferase |
| Aspartate aminotransferase |
| Glomerular filtration rate |
| Albumin |
| Direct bilirubin |
| Thyroid-stimulating hormone |
| White blood cells |
| Erythrocytes |
| Hemoglobin |
| Hematocrit |
| Mean corpuscular hemoglobin |
| Mean corpuscular volume |
| Mean corpuscular hemoglobin concentration |
| Platelets |
| Neutrophils |
| Lymphocytes |
| Monocytes |
| Eosinophils |
| Basophils |
| Vitamin D (at baseline only) |
